# Tracheobronchial amyloidosis and multiple myeloma

**DOI:** 10.36416/1806-3756/e20240080

**Published:** 2024-05-08

**Authors:** Luciana Volpon Soares Souza, Arthur Soares Souza, Edson Marchiori

**Affiliations:** 1. Ultra X, São José do Rio Preto (SP) Brasil.; 2. Faculdade de Medicina de Rio Preto, São José do Rio Preto (SP) Brasil.; 3. Universidade Federal do Rio de Janeiro, Rio de Janeiro (RJ) Brasil.

**Figure 1:**
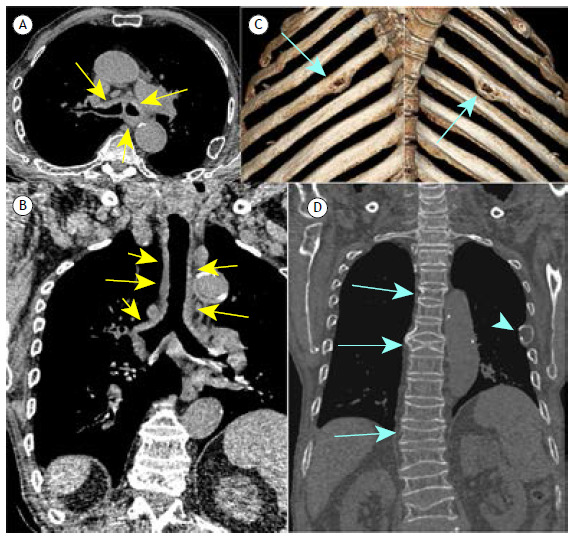
Axial CT of the chest (mediastinal window) at the level of the proximal segment of the bronchial bifurcation (in A) and a coronal reconstruction scan (in B) showing thickening of the tracheal wall and main bronchi (yellow arrows). In C, three-dimensional reconstruction of the chest wall demonstrating bilateral lytic lesions on the ribs (blue arrows). In D, coronal reconstruction showing partial collapse of multiple vertebral bodies (green arrows) and a lytic lesion on a left rib (arrowhead).

An 86-year-old man was admitted with cough and weight loss. He reported episodes of mild hemoptysis and denied fever or other symptoms. He was a smoker (40 pack-years) with a previous history of multiple myeloma. Physical examination demonstrated wheezing. Laboratory test results were unremarkable. Chest CT revealed tracheal and bronchial wall thickening, and lytic lesions on the ribs and vertebral bodies with partial collapse (Figure 1).

The patient was referred for fiberoptic bronchoscopy with BAL. Bronchoscopy showed tracheal and bronchial wall thickening, with swelling and hypertrophy of a brittle, and easily bleeding mucosa, as well as submucosal plaques. BAL fluid was negative for neoplastic cells, bacteria, and fungi. Biopsy of the tracheal walls revealed a stroma occupied by an amorphous eosinophilic material that was positive on Congo red staining and exhibited apple-green birefringence under polarized light, consistent with amyloidosis.

Multiple myeloma and amyloidosis are characterized by abnormal accumulation and deposition of monoclonal plasma cells and extracellular protein fibrils. Multiple myeloma is often complicated with amyloidosis.^1^ ^)^ In the thoracic compartment, amyloidosis typically affects the heart, but it can also involve the pulmonary parenchyma, tracheobronchial tree, and other sites. Pulmonary involvement is rare, and amyloidosis is reported as tracheobronchial, diffuse/alveolar-septal, or nodular.^2^
